# Institutional Notes and News

**Published:** 1911-06-03

**Authors:** 


### INSTITUTIONAL NOTES AND NEWS.

**Further Protest against Cardiff Mental Hospital.**

On Thursday next week a protest meeting will be held to oppose the decision of a section of the authorities of the Cardiff Mental Hospital to build a research house at the expense of the ratepayers, in which experimental research on animals can be carried on. Our previous issue of May 20, page 188, will have led our readers to expect some such further opposition. We understand that Dr. Forbes Winslow, the founder of the British Hospital for Mental Diseases, will be the principal speaker.

**First Results at the Schiff Home.**

The first results obtained by the Schiff Home of Recovery for patients after surgical treatment were laid before the annual meeting last week. Since the opening of the Home during November last, 188 surgical convalescent cases have been received at Chobham from the London hospitals. It will be remembered that the foundation of the Home was due very largely to the gift of £100,000 from Mr. E. F. Schiff, who was in the chair on this occasion. The Chairman of the Home was re-appointed in the person of Lord Lytton, and Sir Richard Martin and the Rev. F. Whyley are again treasurer and honorary secretary.

**The Gordon Hospital Emergency.**

Owing to the unexpected calling-in of a mortgage for payment during May the Gordon Hospital for rectal diseases has found itself compelled to raise a sum of £4,500. This fact being made known in March, an appeal was issued, but the Hon. Arthur G. Davey confessed at last week's annual meeting that it had not been successful. At the present moment, we understand, King Edward's Hospital Fund is considering the matter. The interest, said Mr. Davey, has always been paid regularly. For some months the owners of the mortgage are not expected to adopt extreme measures. Mr. Davey then appealed for a mortgage redemption fund, by which the debt could stand over until it was paid.

**Legacies for the Anti-vivisection Hospital.**

Last week's activity of the anti-vivisectionists over the Cromer episode has attracted additional attention to their hospital's annual meeting, which was held almost simultaneously. In some respects progress is noticeable. During the past year the Board of Trade has approved the incorporation of the institution as the Anti-vivisection and Battersea General Hospital. Some remarkable bequests have been made, which include £4,000 in legacies, and the present of two houses, which will form a most desirable annexe to the hospital. In accordance with the giver's intention, one of these houses will be used for the accommodation of children only. As regards the work in the wards, 215 in-patients were admitted, a decrease of 36, while there appears to have been an increase in the number of out-patients, which rose from nearly seven to over eight thousand. The total income, £2,469, fell short of the total cost of administration, £3,624, by £1,155. The meeting was presided over by the Rev. J. P. Fallowes, the chairman of the Board of Management.

